# *Ps*d1 Effects on *Candida albicans* Planktonic Cells and Biofilms

**DOI:** 10.3389/fcimb.2017.00249

**Published:** 2017-06-09

**Authors:** Sónia Gonçalves, Patrícia M. Silva, Mário R. Felício, Luciano N. de Medeiros, Eleonora Kurtenbach, Nuno C. Santos

**Affiliations:** ^1^Faculdade de Medicina, Instituto de Medicina Molecular, Universidade de LisboaLisbon, Portugal; ^2^Instituto de Biofísica Carlos Chagas Filho, Universidade Federal do Rio de JaneiroRio de Janeiro, Brazil

**Keywords:** antimicrobial peptides, atomic force microscopy, *Candida albicans*, biofilm, confocal microscopy

## Abstract

*Candida albicans* is an important human pathogen, causing opportunistic infections. The adhesion of planktonic cells to a substrate is the first step for biofilm development. The antimicrobial peptide (AMP) *Ps*d1 is a defensin isolated from *Pisum sativum* seeds. We tested the effects of this AMP on *C. albicans* biofilms and planktonic cells, comparing its activity with amphotericin B and fluconazole. Three *C. albicans* variants were studied, one of them a mutant deficient in glucosylceramide synthase, conferring resistance to *Ps*d1 antifungal action. Atomic force microscopy (AFM) was used to assess morphological and biomechanical changes on fungal cells. Surface alterations, with membrane disruption and leakage of cellular contents, were observed. Cytometry assays and confocal microscopy imaging showed that *Ps*d1 causes cell death, in a time and concentration-dependent manner. These results demonstrate *Ps*d1 pleiotropic action against a relevant fungal human pathogen, suggesting its use as natural antimycotic agent.

## Introduction

*Candida albicans* is an opportunistic human pathogen, causing oral, genital and systemic fungal infections, which are especially relevant among immunocompromised patients (Berman and Sudbery, 2002). Despite the available antifungal therapies, mortality and morbidity caused by this pathogen are still high (Behnsen et al., 2008). Candidiasis associated with intravenous lines and bioprosthetic devices is problematic, since these devices can act as substrates for biofilm growth. The presence of biofilms can result in serious problems due to their resistance to antimicrobial agents. This resistance is developed by the presence of quorum-sensing molecules that plays an important role in the biofilm formation and virulence, based on the local density of the fungal population present for the construction and/or dissolution of biofilm communities (Donlan, 2002; Kruppa, 2009; Deveau and Hogan, 2011). There is a thin line between free-floating planktonic cells and biofilm growth. In fact, biofilm development begins when planktonic cells adhere to the substrate. Adhered/adherent cells grow and divide, creating a protective matrix including secreted exopolysaccharides (EPSs) (Donlan, 2002; Kruppa, 2009; Deveau and Hogan, 2011). EPSs contribute to the volume of a biofilm, and for its slimy macroscopic properties. A fully developed biofilm is highly structured, with layers of cells rising up and permeated by fluid-filled microchannels (Donlan, 2002). These dynamic communities can spread across surfaces, incorporate particulates and other microbes from the surrounding environment, and continually shed new planktonic cells (Stephens, 2002). *C*. *albicans* has the ability to attach, colonize, and form biofilms on a variety of surfaces. The importance of *C. albicans* as a pathogen has led to a significant effort on the development of new strategies to control and detect the disease (Srinivasan et al., 2011).

Fungi possess a unique cell wall and cell membrane that can serve as targets for antifungal agents. The fungal cell membrane is similar to other eukaryotic cells, composed of a lipid bilayer with proteins embedded within it, having ergosterol as its main sterol (Katzung et al., 2011). Glycosphingolipids (GSL) are a family of lipids that act as key components of biological membranes in animals, plants and fungi (Leipelt et al., 2001; Halter et al., 2007; Daniotti and Iglesias-Bartolome, 2011). The most common GSL found in fungi is glucosylceramide (GlcCer), present in the cell membrane of most fungi, such as *Pichia pastoris, C. albicans, Cryptococcus neoformans, Aspergillus fumigatus, Sporothrix schenckii*, and *Neurospora crassa* (Barreto-Bergter et al., 2004; Saito et al., 2006). Large amounts of this glycosphingolipid have also been found in the fungal cell wall (Nimrichter and Rodrigues, 2011). Its functions during fungal growth/dimorphism have been correlated with the virulence process (Rittershaus et al., 2006), suggesting GSL as potential targets on the development of new antifungal drugs (Rittershaus et al., 2006; Nimrichter and Rodrigues, 2011; Gonçalves et al., 2012).

Antimicrobial peptides (AMPs) are cationic molecules characterized by short sequences (usually 15–50 amino acid residues), which possess both hydrophobic and hydrophilic residues, resulting in amphipathic structures. Endogenous AMPs from plant, fungal or animal origin are produced in order to protect themselves from pathogenic microbes. This adaptive mechanism makes them essential to the innate immune system. AMPs therapeutic activity unfolds against bacteria, fungi, protozoan and metazoan parasites, viruses, skin diseases and tumor cells (Li et al., 2012; Morizane and Gallo, 2012; Torrent et al., 2012). Extensive information on their therapeutic activity and mode of action has been given elsewhere (Silva et al., 2014). These natural antibiotics have the additional advantage of not being prone to the development of antibiotic-resistant microbial strains (Korting et al., 2012).

*Ps*d1 is a cysteine-rich 46 amino acid residues defensin, isolated from the seeds of the garden pea (*Pisum sativum*) (Almeida et al., 2000, 2002; Cabral et al., 2003; de Medeiros et al., 2010). It is found primarily in epidermal tissues and vascular bundles of pea pods. This peptide exhibits high antimicrobial activity against several filamentous fungi and the dimorphic *C*. *albicans* and *N*. *crassa*, but not against several tested bacteria (Almeida et al., 2000, 2002; Lobo et al., 2007; de Medeiros et al., 2010). *Ps*d1 at 20 μM has been shown to cause a 100% growth inhibition of *C. albicans* wild type (WT), while having a 70% inhibition of its corresponding *C. albicans gcs*-deleted strain (Δ*gcs*) (de Medeiros et al., 2014). Recently, we demonstrated through membrane partition studies that *Ps*d1 has high affinity and specificity for membranes with ergosterol and GlcCer, as in fungal cells (Gonçalves et al., 2012). On the contrary, this defensin has a low interaction with cholesterol-rich membranes, explaining the reduced toxicity of *Ps*d1 to human cells (Gonçalves et al., 2012). A lack of *Ps*d1 internalization in *C*. *albicans* mutant strain (Δ*gcs*) has been shown by confocal microcopy (de Medeiros et al., 2014). Together, these results indicated that GlcCer is important for *Ps*d1 interaction with the fungal plasma membrane, as well as for its internalization.

In the present study, the mode of action of *Ps*d1 was assessed through the evaluation at the nanoscale level of its effects on the cell morphology, roughness and stiffness of three different *Candida albicans* strains. Differences between planktonic cells and biofilms were found for the variants studied. Confocal microscopy and atomic force microscopy (AFM) images of untreated and treated *C*. *albicans* cells showed that *Ps*d1 kills planktonic cells at 20 μM, while total inhibition and partial eradication of biofilm were only observed at a 10-fold higher concentration. The *C*. *albicans* Δ*gcs* mutant showed alterations in cell morphology and roughness even in the absence of the peptide, both for biofilms and planktonic cells. In the presence of *Ps*d1, adherence of planktonic cells was decreased and a total inhibition and/or eradication of the biofilm were observed. These results demonstrate several key aspects for *Ps*d1-fungal membrane interaction, for which GlcCer is highly relevant. Additionally, our data indicates that the defensin has a pleiotropic action, with an additional component of its antimicrobial action occurring intracellularly.

## Materials and methods

### *Candida albicans* cultures preparation

Three *C*. *albicans* strains were studied: a clinical isolate (CI) collected from a patient at the Santa Maria Hospital (Lisbon, Portugal), SC5314/ATCC MYA-2876 (WT) and SC5314 CAI4 *ura3*Δ*::imm434/ura3*Δ*::imm434* (Δ*gcs*), congenic to SC5314, kindly provided by Dr. Dirk Warnecke (Institut fur Allgemeine Botanik, University of Hamburg, Germany). On Δ*gcs*, the glucosylceramide synthase gene (HSX11) was disrupted, making the strain deficient on glucosylceramide lipid (Leipelt et al., 2001). Strains stocks were kept at −80°C, with 15% glycerol. Cells in stock, previously thawed, were inoculated onto Yeast Peptone Dextrose (YPD, Sigma Aldrich, USA) agar plates, and incubated for 48 h at 37°C. After this period, an isolated fungal colony was cultured overnight at 25°C with shaking at 180 rpm in YPD broth. During this period, the culture reaches the stationary phase of growth. Cells were harvested by centrifugation at 1,880 *g* for 10 min at 4°C, the supernatant was removed and cells were washed three times with 10 mM HEPES buffer pH 7.4 with 150 mM NaCl, for planktonic studies, and with 10 mM phosphate buffered saline (PBS, 2.7 mM potassium chloride, 137 mM sodium chloride) pH 7.4 for biofilm assays. Afterwards, cell concentration was determined and the initial suspension was diluted to the concentration necessary for each experiment.

### Susceptibility of planktonic *C. albicans* to amphotericin B, fluconazole and *Ps*d1

*In vitro* antifungal susceptibility tests were performed to determine the minimal inhibitory concentration (MIC). It was determined according to recommendation of the National Committee for Clinical Laboratory Standards (National Committee for Clinical Laboratory Standard, 1997), by the microdilution method, in 96-well microplates (Brito et al., 2010; Eksi et al., 2013). RPMI 1640 medium with L-glutamine (Gibco-Life Technologies, UK) was used, supplemented with 0.2% glucose, 165 mM MOPS (3-morpholinopropanesulfonic acid) (AppliChem, Germany) and buffered to pH 7.4. For the sake of comparison and as positive control, the conventional antifungal drugs amphotericin B (AMPH B) and fluconazole (FCZ) (Sigma Aldrich, USA) were also tested against the selected strains. Each well was prepared to a total volume of 200 μl, with the growth medium (RPMI), different AMPH B or FCZ concentrations (0.001 to 100 μg/ml) and *C*. *albicans* (2 × 10^3^ cells/ml). Controls without antifungal were also tested. Plates were incubated for 48 h at 37°C, after which optical density was measured at 540 nm. MIC was defined as the minimal concentration of a drug that, after incubation, causes 100% growth inhibition of an organism (Andrews, 2001). Experiments were performed in triplicate and values were analyzed with GraphPad Prism 5, using the Gompertz equation for MIC determination (Lambert and Pearson, 2000). Experiments with *Ps*d1 were performed at 90 μg/ml (20 μM) (de Medeiros et al., 2014).

### Biofilm development and XTT/menadione testing assay

The formation and susceptibilities of *C*. *albicans* biofilms were determined by 96 well-plate based method (Pierce et al., 2008). To determine the optimal cell concentration for biofilm formation for each strain, 100 μl *C*. *albicans* suspension cells in RPMI 1640 with glucose 2% and 165 mM MOPS, at 1.0 × 10^5^, 1.0 × 10^6^, 1.0 × 10^7^, and 1.0 × 10^8^ cells/ml were placed on a polystyrene 96 well plate, each sample in triplicate. Plates were incubated for 12, 24, 48, 72, and 96 h at 37°C, in order to establish the optimal cell concentration for biofilm development. At the end of each step-time (incubation time), the biofilm was washed three times with PBS to remove planktonic and/or no adherent cells. At this point, 100 μl of XTT/menadione solution (1 μl menadione 1 mM in 10 ml XTT 0.5 g/l) were added on each well-plate (where the biofilm is formed) and incubated for 2 h at 37°C. After this time, an orange color reveals the metabolic activity of the cells within the biofilm. The supernatants were transferred to a new plate and the optical density measured at 490 nm.

### Atomic force microscopy imaging

Planktonic cells imaging were performed for all *C. albicans* strains as follows, 1 × 10^5^ cells/ml were incubated at 25°C in HEPES buffer, with agitation, for 6 and 24 h. AMPH B, FCZ, and *Ps*d1 final concentrations were equal to the MIC and 10-fold higher than the MIC. As a control, cells without antifungal treatment were used. A 100 μl droplet of each test sample was applied onto a poly-L-lysine (PLL)-coated coverslip and left at 25°C for 2 h. After deposition, the samples were rinsed 10 times with filtered (0.2 μm) deionized water and air-dried at 25°C.

Untreated and treated cells were imaged using a JPK NanoWizard II atomic force microscope (JPK Instruments, Berlin, Germany) mounted on a Zeiss Axiovert 200 inverted microscope (Carl Zeiss MicroImaging, Jena, Germany). Measurements were carried out in intermittent contact mode, at room temperature, using uncoated silicon ACL cantilevers (Applied NanoStructures, Mountain View, CA, USA). These cantilevers have typical resonance frequencies of 145–230 kHz and spring constants of 20-90 N/m. The scan rate was set to less than 1 Hz for imaging and image resolution was set to 512 × 512 pixel for all images. Height, error signal and phase contrast images were recorded, and line-fitted as required. From recorded images, height and size information was obtained with the JPK Data Processing software v.4.2.53.

Roughness analysis of AFM height images was performed using the Gwyddion 2.31 software (Czech Metrology Institute, Brno, Czech Republic). Roughness was calculated from the root mean square value (RMS, i.e., standard deviation of the distribution of heights over a 1 × 1 μm^2^ imaged area). The results of this processing were statistically analyzed using analysis of variances (ANOVA) and Bonferroni post-tests.

### AFM-based cell stiffness measurements

*C. albicans* washed suspensions were incubated at room temperature, with agitation, for 24 h. AMPH B, FCZ, and *Ps*d1 final concentrations were 10-fold higher than the MIC. Final cell concentration was 1 × 10^5^ cells/ml and as control, cell samples were incubated without any treatment. 100 μl of each sample were placed onto a poly-L-lysine (PLL)-coated glass coverslip and left at 25°C for 2 h. After deposition, samples were rinsed 10 times with HEPES buffer to remove any cells that had not adhered to the coverslip. 100 μl of HEPES buffer were added to the adhered cells to avoid sample drying. Measurements were carried out in HEPES buffer at 25°C, using 200 μm long gold reflex coated silicon-nitride OMCL-TR400PSA-1 cantilevers (Olympus, Japan). These cantilevers have typical resonance frequencies of 8–14 kHz and spring constants of approximately 0.02 N/m.

First, to have a prior overview of the cells shape and height, force maps were performed using a 10 μm/s approach and retraction speed, Z length of 3 μm and a relative set-point of 0.4 V. The coordinates on the map were then chosen. Afterwards, one location per each cell was chosen and force-distance measurements were conducted over those coordinates, in triplicate, using a 3 μm/s approach and retraction speed, Z length of 3 μm and a relative set-point of 0.4 V. These conditions ensure that the identation ranged from 5 to 10% of cells height. Retraction force-distance curves were processed with the JPK Data Processing software v. 4.2.53. After processing, the four-sided pyramid Hertz modified equation was applied to the curves and the Young's modulus obtained. The results of this processing were statistically analyzed using ANOVA and Bonferroni post-tests.

### Biofilm inhibition and eradication assays

Biofilm inhibition and eradication assays were determined by using the cells conditions obtained in biofilm development assays. Once the cells concentrations for each strain were determined, concentrations of AMPH B, FCZ, and *Ps*d1 used for biofilm inhibition and eradication assays correspond to 10 and 100-fold higher than the MIC. In inhibition assays, the antifungal drugs were placed at the same time than cells (pre-mixing antifungal with cells) and incubated during the same step-time previously determined for biofilm development. For eradication assays, antifungals were added once the biofilm was formed, for each strain, and left to incubate for 24 h at 37°C. In both cases, biofilms medium were replaced with PBS for microscopy measurements. For AFM imaging, biofilms were washed 10 times with filtered deionized water (0.2 μm) and air-dried at room temperature. Biofilm images for formation, inhibition and eradication of *C*. *albicans* strains were measured directly on the surface where cells were grown.

### Live/Dead measurements of biofilms and plancktonic cells by confocal microscopy and flow cytometry

Live/Dead assay kit was used both on *C. albicans* biofilms and plancktonic cells. LIVE/DEAD® *Funga*Light™ Yeast Viability Kit (L34952, LifeTechnologies, USA) is composed by two fluorescent probes, SYTO 9 and propidium iodide (PI). In a population of live and dead cells, SYTO 9 nucleic acid labels all yeast in a population, those with intact membranes and those with damaged membranes. In contrast, PI penetrates only yeast with damaged membranes, causing a reduction in the SYTO 9 stain fluorescence by fluorescence resonance energy transfer (FRET) when both dyes are present (Johnson and Spence, 2010). As a result, yeast with intact membranes is stained with green fluorescence, whereas yeast with damaged membranes is stained with red fluorescence.

Optical microscopy experiments with a Zeiss LSM 510 META confocal point-scanning microscope (Jena, Germany) were carried out in order to examine the architecture and the viability of the cells before and after exposure to antifungal agents. Argon (488 nm; 45 mW) and diode-pumped solid-state (561 nm; 15 mW) lasers were used with a 40 × dry-objective. Cells were incubated with AMPH B (10 mg/ml), FCZ (40 mg/ml), or *Ps*d1 (900 μg/ml) for 24 h prior to imaging. These antifungal concentrations were selected to be 10 times more than the planktonic MIC. Afterwards, biofilms were labeled with SYTO 9 and PI probes, incubated for 15 min and images acquired. Images were analyzed with ImageJ 1.47v (rsbweb.nih.gov/ij/).

For flow cytometry assays, *C. albicans* strains were incubated for 24 h with AMPH B, FCZ, and *Ps*d1 with antifungal concentration equal to the planktonic MIC and 10-folder higher. The double labeled cells were considered as positive result for death, since the green dye is present in all cells, and besides the FRET phenomena, the green fluorescence intensity had always a small contribution. *C. albicans* stains were performed according to the manufacturer's instructions. Briefly, 1 × 10^5^ cells/ml in HEPES buffer were stained with both dyes to a final concentration of 3.34 and 20 μM (SYTO 9 and PI, respectively). All samples were kept at room temperature in the dark for 15 min before flow cytometry analysis. Experiments were performed in a BD Accuri C6 Flow Cytometer (BD Biosciences, San Jose, CA, USA), using blue (488 nm) and red (640 nm) lasers to excite stained cells. Green fluorescence emission was detected with a 530 nm bandpass filter and red fluorescence emission was detected with a 670 nm bandpass filter. Fluorescence emission was acquired in bioexponential scale, and data were collected for 40 000 cells. All flow cytometer results were analyzed using FlowJo Software version 10.0x (Tree Star Inc., Ashland, OR, USA).

## Results

### Susceptibility of planktonic *C. albicans* to AMPH B, FCZ, and *Ps*d1

The activity of AMPH B and FCZ against the three *C. albicans* strains was determined by measuring their susceptibility to the antifungal drugs, as shown in Figure S1 (Supplementary Material). The obtained curves were fitted and MIC values were obtained using the Gompertz equation (Lambert and Pearson, 2000). The values used for *Ps*d1 were reported in previous works (20 μM) (de Medeiros et al., 2014). As shown in Figure S1, MIC values for AMPH B and FCZ differed depending on the strain studied. Altogether, the three *C. albicans* strains were sensitive to AMPH B, FCZ, and *Ps*d1. AMPH B and FCZ showed similar MIC in WT and Δ*gcs* planktonic cells (these four MICs are all on the 3.7–4.0 μg/ml range). Lower values were obtained when CI was tested.

### Biofilm development and XTT/menadione testing assay

Biofilm formation is dependent of cellular adherence to the growth surface. To study the mode of action of *Ps*d1 on *C*. *albicans* biofilm formation, polystyrene surfaces were selected. To optimize the conditions for biofilm formation, XTT assays were performed for the three *C*. *albicans* strains studied. The time and the cell concentrations that ensure the best biofilm growth are shown in Figure S2 (Supplementary Material). As it can be seen, the cell density needed for biofilm development differs from strain to strain. WT and Δ*gcs* need a cell concentration 10-fold higher than the CI (10^5^ cells/ml) to initiate biofilm development. Despite this, the clinical isolate and WT formed biofilm after 24 h, while the mutant strain needs 72 h to ensure biofilm development and adherence.

### AMPH B, FCZ, and *Ps*d1 cause morphological alterations in *C. albicans* plancktonic cells

**Figure 1:**
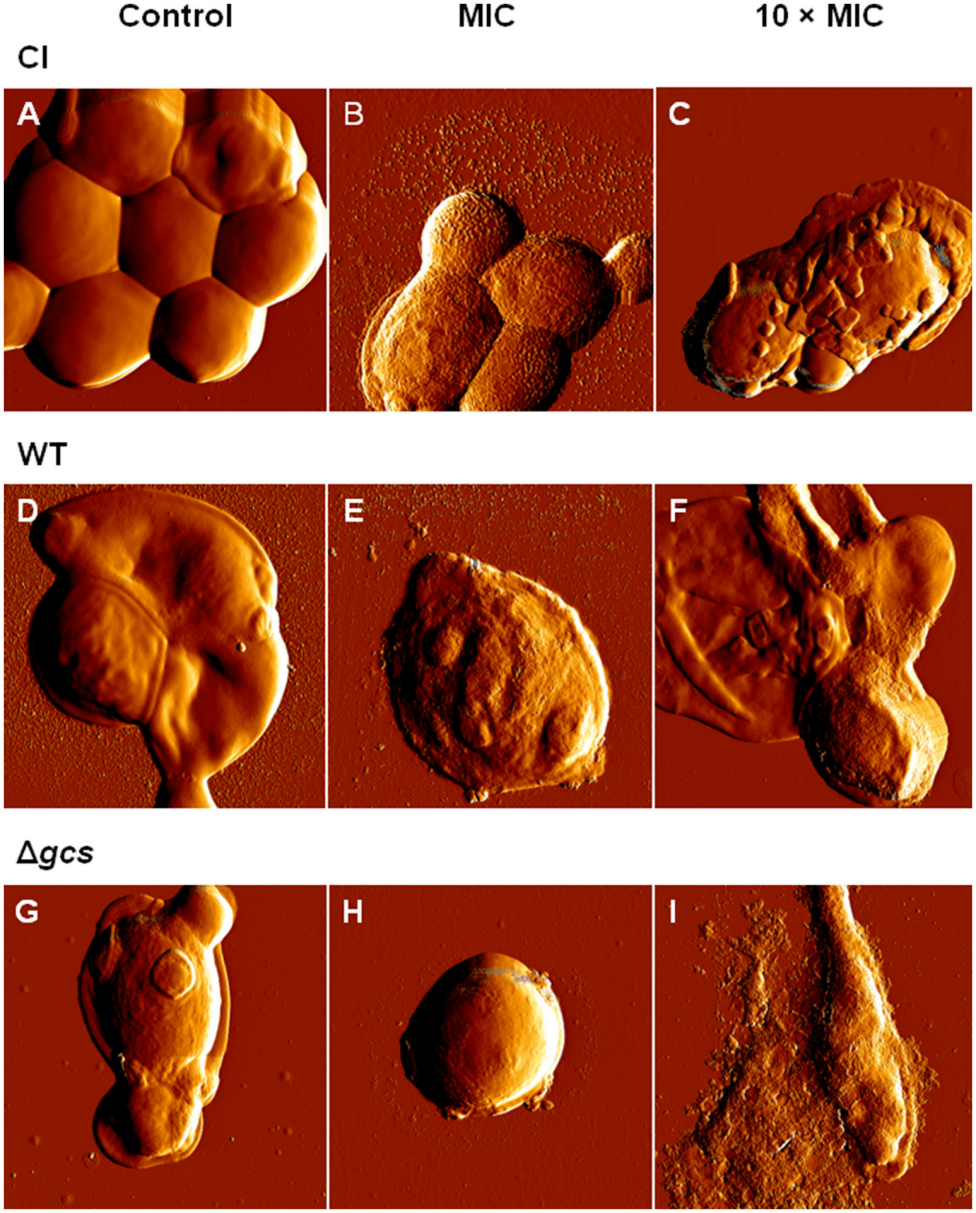
Effect of *Ps*d1 concentration on *C. albicans* planktonic cells. AFM error signal images of clinical isolate **(A–C)**, wild type **(D–F)** and Δ*gcs* **(G–I)** cells after 24 h incubation with *Ps*d1: **(A,D,G)** 0 μM (control); **(B,E,H)** at the MIC (20 μM) and **(C,F,I)** at a 10-fold higher concentration. All images are 10 × 10 μm^2^.

AFM imaging on intermittent contact mode was used to evaluate the effects suffered by *C. albicans* planktonic cells after 6 h and 24 h of incubation with AMPH B, FCZ or *Ps*d1 at the MIC and at 10-fold higher concentrations. The error signal is the difference between the detector signal and the setpoint, and provides images with greater spatial detail. In general, antimicrobial treatments induced morphological changes in all cells. The severity of these effects increased in a time-dependent manner. After 6 h, it was possible to observe small irregularities in the cell surface (blebs) and small vesicles deposited over and around the cells at 10-fold higher than the MIC antifungal concentration (data not shown). As seen in Figure 1 for CI and WT strains, untreated cells (controls) have a smooth surface and regular shape (Figures 1A,D). In contrast with CI and WT, untreated Δ*gcs* cells showed irregular surface (Figure 1G). The incubation for 24 h with *Ps*d1 at MIC concentration showed some deformations at cell surface (Figures 1B,E,H). At 10 × MIC enhanced effects than those obtained at MIC were observed (Figures 1C,F,I). Small blebs are being released from the cells accompanied and internal content released (Figure 1 and Figure S7).

Unlike AMPH B and FCZ (Figures S5, S6), *Ps*d1 effects appear to be more severe. The consensual outcome is the release of the cell internal content or cells completely covered by blebs. For WT treated cells, the height of a bleb is approximately 14 nm (Figure S3). Blebs accumulate over each other, forming a bulk structure on top of the cell. Similar results were obtained for mutant cells (Figure S4). Only for CI cells a peel-off-like morphology was observed at *Ps*d1 10 × MIC (Figure 1C). Of the three treatments, AMPH B is the one that induced a more extensive cell deformation: cells lose volume and membranes appear rougher. The clinical isolate seems to be less affected by FCZ, at both times of incubation and concentration of drug used, only with some irregularities in the cell surface appearing after 24 h of incubation.

### *C. albicans* suffers an increase in surface roughness after treatment with AMPH B, FCZ, or *Ps*d1

**Figure 2:**
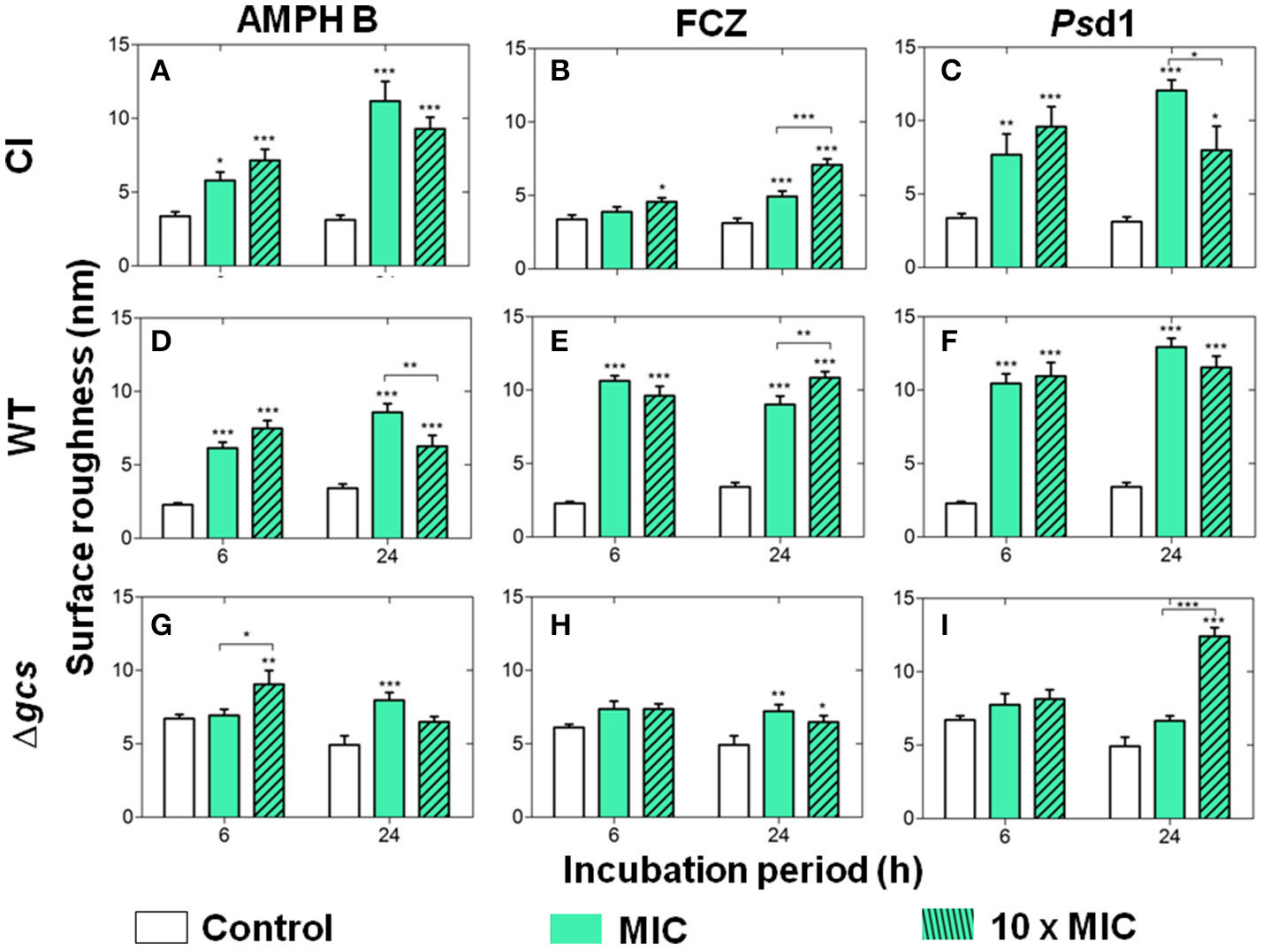
Cell roughness (RMS) measurements after incubation of the clinical isolate **(A–C)**, wild type **(D–F)** and Δ*gcs* **(G–I)** *C. albicans* strains with AMPH B **(A,D,G)**, FCZ **(B,E,H)**, and *Ps*d1 **(C,F,I)**, at the MIC and at a 10-fold higher concentration. Measurements were obtained from AFM height images, on 1 × 1 μm^2^ crops over the cell (*N* = 10). Columns correspond to the mean ± standard deviation of three independent measurements for each cell on a total of 30 cells for each experimental condition. Two-way ANOVA and Bonferroni post-test were performed (^*^p < 0.05; ^**^p < 0.01; ^***^p < 0.001). Error bars indicate the SEM.

Surface roughness was evaluated for the three *C*. *albicans* strains before and after treatment with AMPH B, FCZ, and *Ps*d1 (Figure 2), both after 6 h and 24 h of cell treatment. There was an overall increase of the average roughness upon any of the treatments performed with the planktonic cells. These results, obtained with the RMS formula applied to 1 × 1 μm^2^ images of the surface of the cells, are in agreement with the previous qualitative observations that surface roughness increases after antifungal treatment (Figure 1). Regarding the control conditions, CI and WT cells have lower surface roughness values (Figures 2A,D, control), below 5 nm, whereas Δ*gcs* cells present surface roughness values above 5 nm (Figure 2G, control). WT cells were the most affected (Figures 2D–F) and Δ*gcs* the least affected (Figures 2G–I) by any of the treatments. All treatments with AMPH B caused a statistically significant increase of membrane roughness on CI and WT cells (Figures 2A,D). This was not always the case for Δ*gcs* cells, as their increase in roughness was not statistically significant (Figures 2H,I). When comparing the three cells treated with FCZ (Figures 2B,E,H), less effects on surface roughness were observed for CI and Δ*gcs*. On the contrary, WT cells were strongly affected by this treatment and all conditions tested resulted in a statistically significant increase in surface roughness. Finally, *Ps*d1 increased CI cells roughness in a way similar to AMPH B (Figure 2C), whereas for WT cells the effects of *Ps*d1 (Figure 2F) were similar in magnitude to those of FCZ. Again, as it was observed for AMPH B and FCZ, Δ*gcs* were the least affected by *Ps*d1; yet, the defensin was able to strongly increase the surface roughness of these cells after 24 h of incubation, with a peptide concentration 10-fold higher than the MIC (Figure 2I).

### *C. albicans* loses stiffness after treatment with AMPH B, FCZ, or *Ps*d1

**Figure 3:**
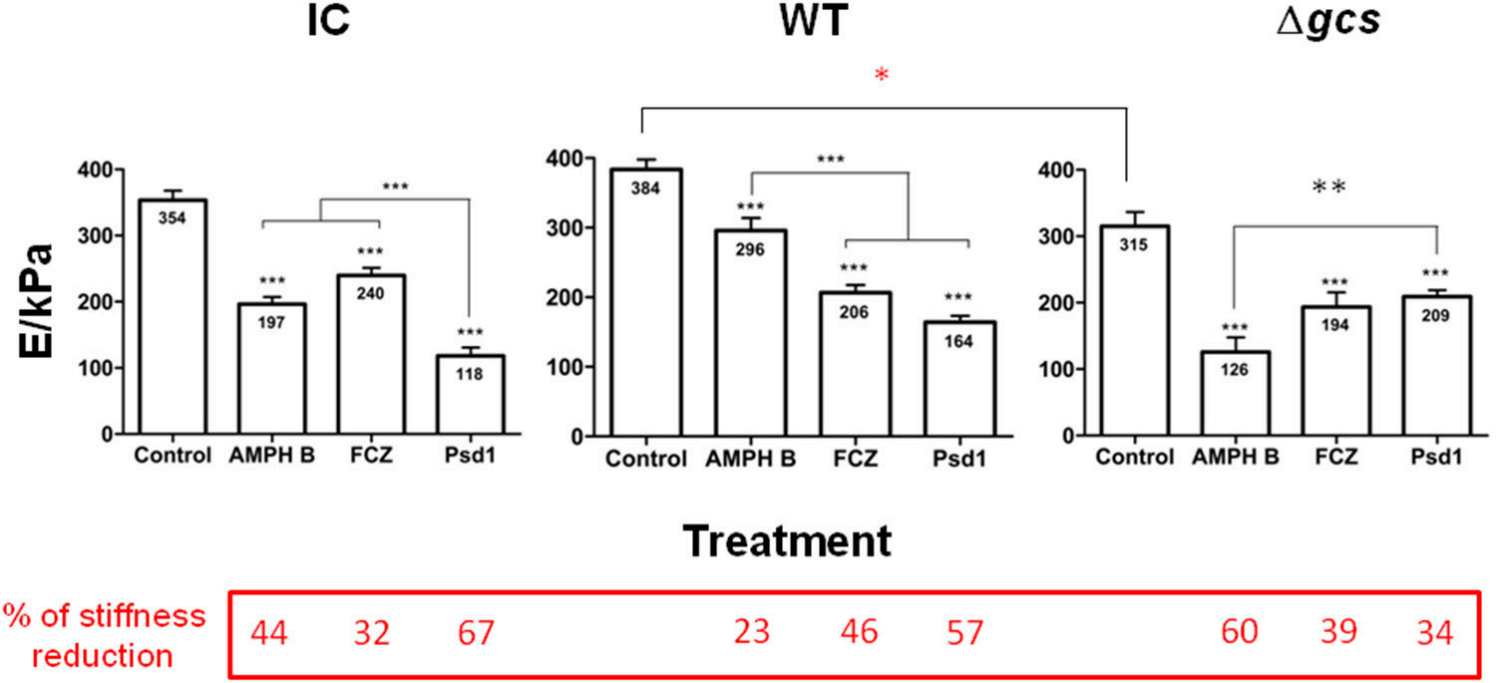
Cell stiffness measurements (Young's modulus calculated from the AFM force-distance curves) after 24 h incubation of *C. albicans* with AMPH B, FCZ, and *Ps*d1 (concentrations 10-fold the MIC). Measurements correspond to triplicate for each cell on a total of 30 cells for each experimental condition. Two-way ANOVA and Bonferroni post-test were performed (^*^p < 0.05; ^**^p < 0.01; ^***^p < 0.001). Error bars indicate the SEM.

Changes in the stiffness of the cells were assessed in two different ways. One was based on the determination of the Young's modulus of the membrane, using AFM-based force spectroscopy (for the three treatments; Figure 3); and the other was based in the observation of AFM phase-contrast images of the cell surface, which allow to visualize and distinguish softer and stiffer areas in the membrane (for *Ps*d1 only; Figure S7).

Young's modulus determination was performed after 24 h of incubation with an AMPH B, FCZ or *Ps*d1 concentration 10-fold higher than the MIC (Figure 3). CI cells had a mean value of membrane stiffness of 354 ± 14 kPa, WT had a mean value of 384 ± 14 kPa and Δ*gcs* had a mean value of 315 ± 21 kPa. When Δ*gcs* cells stiffness was compared with WT, there was an 18% reduction (red asterisk in Figure 3; ^*^p < 0.05). The percentages of stiffness reduction relative to the control were calculated to better understand the different impacts of each treatment (values in the dark red box on the bottom of Figure 3). AMPH B effects on cell stiffness were more severe for Δ*gcs* cells, with a 60% reduction of the cell initial stiffness (Figure 3). WT cells were the less affected by this treatment, with a 23% reduction of the initial stiffness. FCZ was the treatment with the least effect on the three strains and its highest reduction on stiffness was registered for the WT cells, where FCZ caused a reduction to nearly half of the initial stiffness. From the three treatments, *Ps*d1 caused the largest reduction on CI (67%) and WT (57%) cells stiffness, whereas for Δ*gcs* cells this was the treatment with a lower effect (34% stiffness reduction). In all cases, the treatment with the antifungal drugs, including *Ps*d1, lead to statistically significant decreases on the cells stiffness (Figure 3; *p* < 0.001).

By phase contrast imaging of *C. albicans* after *Ps*d1 treatment, it was possible to distinguish some changes related to sample properties such as stiffness and softness (Magonov et al., 1997; Martinez and Garcia, 2006; Garcia et al., 2007; Nie et al., 2011). Phase contrast images of control cells of the three strains all present a homogenous surface. For the three strains, the results observed are roughly the same. Blebs in the cell surface caused by the treatment with the peptide are softer than the surrounding cell surface (Figure S7, phase contrast images in Supplementary Material), and even when there are no blebs formed, these images allow to distinguish softer and stiffer areas.

### *Ps*d1 provoke death in *C. albicans* planktonic cells

Flow cytometry experiments were carried out in order to determine if *Ps*d1 kills planktonic *C. albicans* cells, by live/dead staining. CI, WT, and Δ*gcs* cells were incubated for 24 h with a concentration of AMPH B, FCZ or *Ps*d1 equal to the MIC and 10-folder higher. The quadrants (unstained, live, dead and double positive cells) were established for each strain using their respective controls (data not shown).

**Figure 4:**
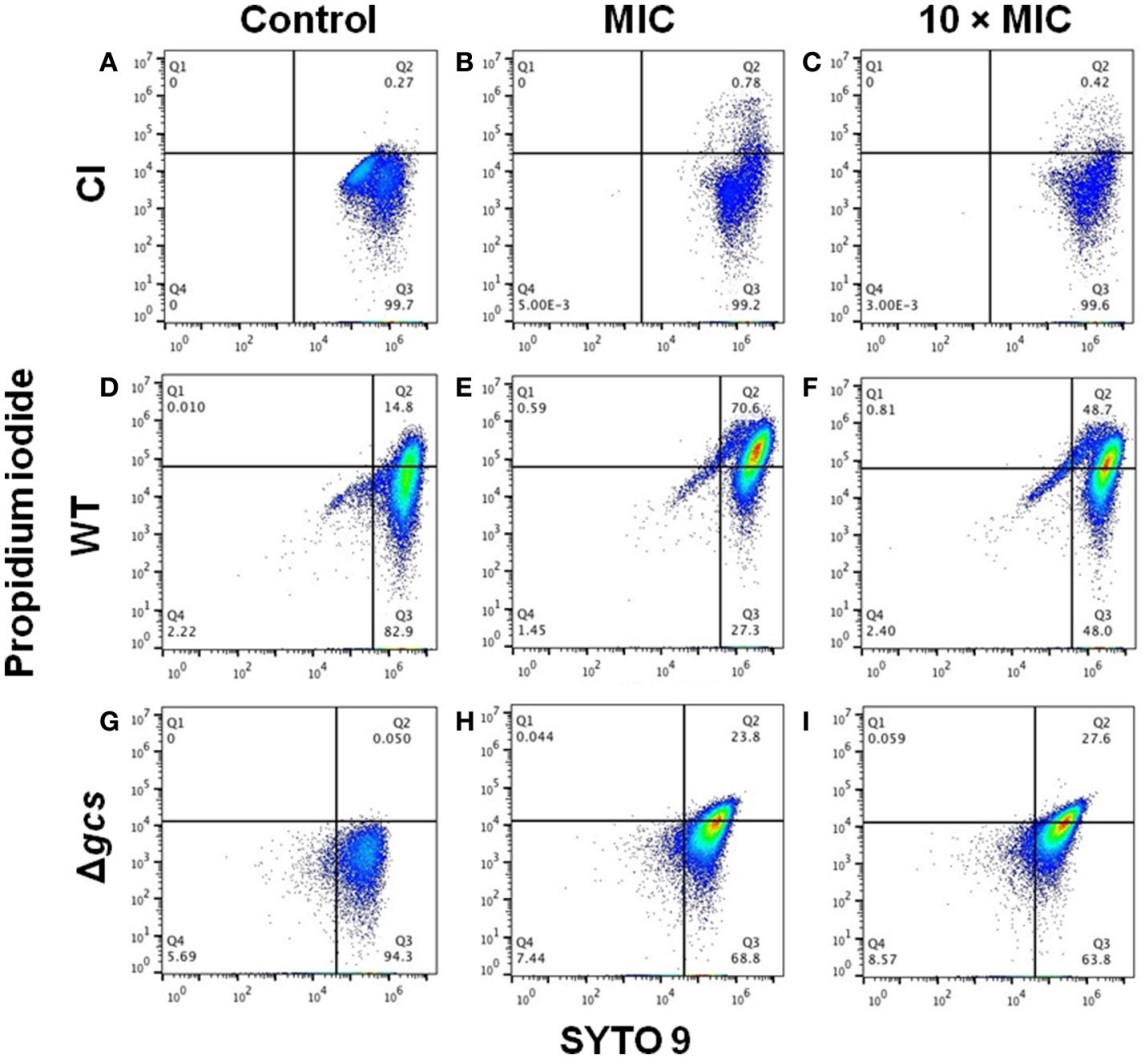
Flow cytometry dot plots of clinical isolate **(A–C)**, wild type **(D–F)** and Δ*gcs* **(G–I)** planktonic cells after treatment with *Ps*d1. Cells stained with both dyes after 24 h incubation with *Ps*d1 at MIC concentration (20 μM) **(B,E,H)** and 10-folder higher (200 μM) **(C,F,I)**, as well as in its absence (**A,D,G**, control).

Figure 4 shows the flow cytometry dot plots obtained for CI, WT, and Δ*gcs* planktonic cells after treatment with *Ps*d1 at MIC and 10-folder higher. Here, *Ps*d1 had less of an effect on CI cells, with fewer cells dead in the presence of the peptide (Figures 4A–C). For WT and Δ*gcs*, the percentage of dead cells increased to 70.6% and 23.8% in presence of *Ps*d1 at MIC concentration, respectively. Curiously, when peptide concentration was increased to 10-folder higher, the percentage of dead cells seems to be similar for Δ*gcs* cells (27.8%), but not for WT cells (48.7%).

Comparing all these results with those obtained for AMPH B and FCZ (Figures S8–S10), it is possible to infer that *Ps*d1 had a stronger effect in WT and Δ*gcs* cells (Figures 2E,F,H,I, respectively) similar to AMPH B (Figures S9, S10). AMPHB and FCZ antifungals had the same effect in CI cells: approximately 20% of cells were killed by their effect (Figures 4B,C, and Figure S8). *Ps*d1 and AMPH B had the same behavior in all three strains: less cell death was induced in CI strain by antifungal action, while increasing death was observed in WT and Δ*gcs* cells. On the contrary, FCZ showed less dead cells (less than 30%) in *C. albicans* strains.

### AFM imaging of *C. albicans* biofilms

**Figure 5:**
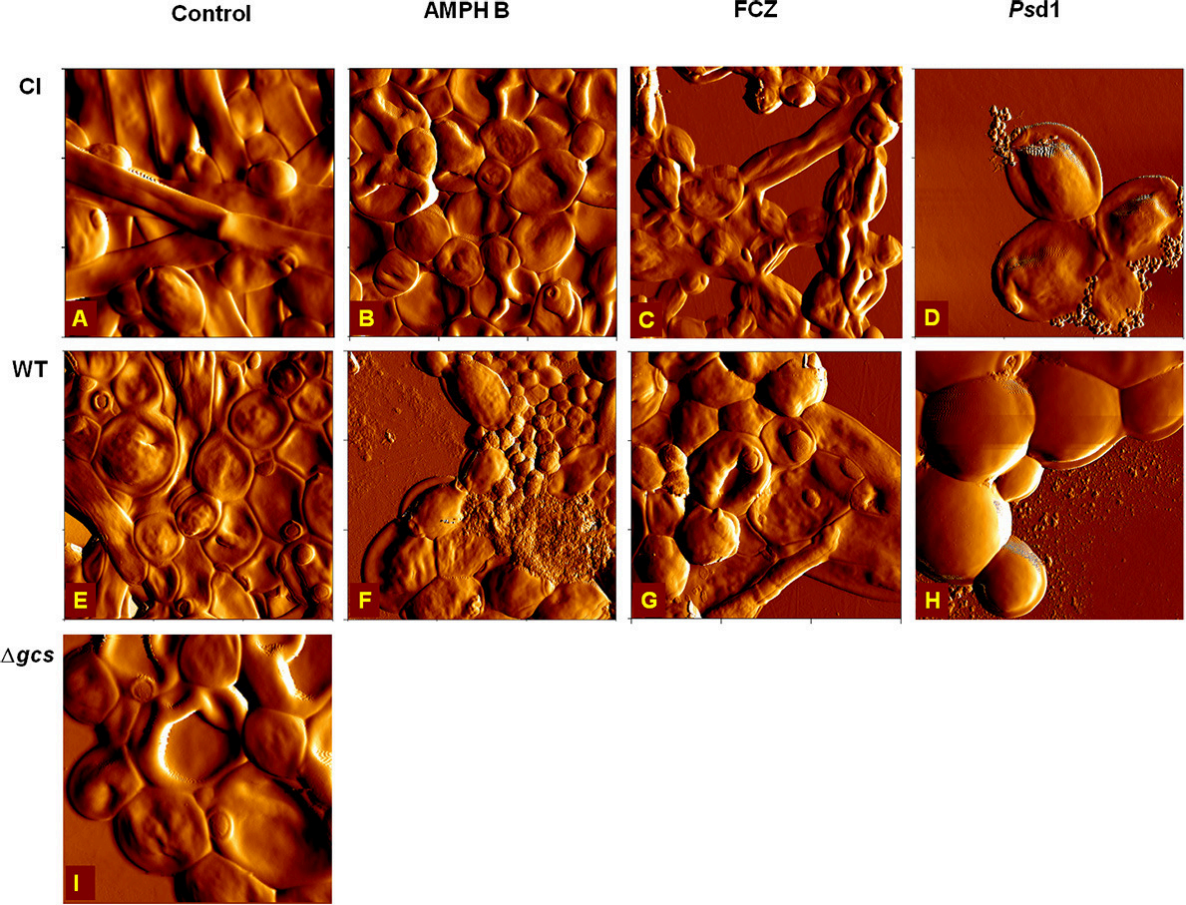
AFM error images of *C. albicans* strains eradication assays. Clinical isolate **(A–D)**, wild type **(E–H)** and Δ*gcs* **(I)**, in the absence of AMPH B **(B,F)**, FCZ **(C,G)** and *Ps*d1 **(D,H)** at 10 × MIC. No image of antifungal treatment of Δ*gcs* biofilm with AMPH B, FCZ, and/or *Ps*d1 is shown, as the peptide eradicates the formed biofilm. All images are 15 × 15 μm^2^, except for **(D,H,I)**, which are 10 × 10 μm^2^.

AFM was used for imaging the effect of AMPH B, FCZ, and *Ps*d1 on *C*. *albicans* biofilms after incubation for 24 h at a concentration 10 times higher than the MIC for planktonic cells (Figure 5). Inhibition of biofilm development was observed for CI and WT strains when treated with AMPH B, FCZ or *Ps*d1 (data not shown). The inhibition of biofilm development showed absence of hyphae or pseudohyphae. Budding yeast cells in small groups (4–8 cells) were observed for all antifungal treatments. In the presence of AMPH B, CI seems to be less affected when compared to WT. Images of the CI biofilm (Figure 5B) show an absence of both pseudohyphae and hyphae. The effect of AMPH B was more evident on WT biofilm (Figure 5F): large deformed cells together with cells of reduced size. Both for the CI and WT, the presence of surface uncovered by the cells evidences changes in the biofilm biomass upon AMPH B action. These effects were more remarkable with FCZ and *Ps*d1. The uncovered surface area was increased and the loss of volume was observed for CI and WT strains. FCZ at 10 × MIC induced a decrease on cell volume and size (Figures 5C,G). *Ps*d1 caused important morphological changes on the cell surface. For the CI treated with *Ps*d1 at 10 × MIC, the appearing of blebs and the loss of cell volume were observed (Figure 5D). WT seemed to be less affected by *Ps*d1. Nevertheless, the appearing of small blebs can be noticed (Figure 5H). The percentages of live and dead cells were experimentally quantified (Table S1). As shown, AMPH B is more effective on biofilm eradication, when compared to FCZ and *Ps*d1.

### Live/Dead cell confocal imaging of *C. albicans* biofilms–inhibition and eradication assays

**Figure 6:**
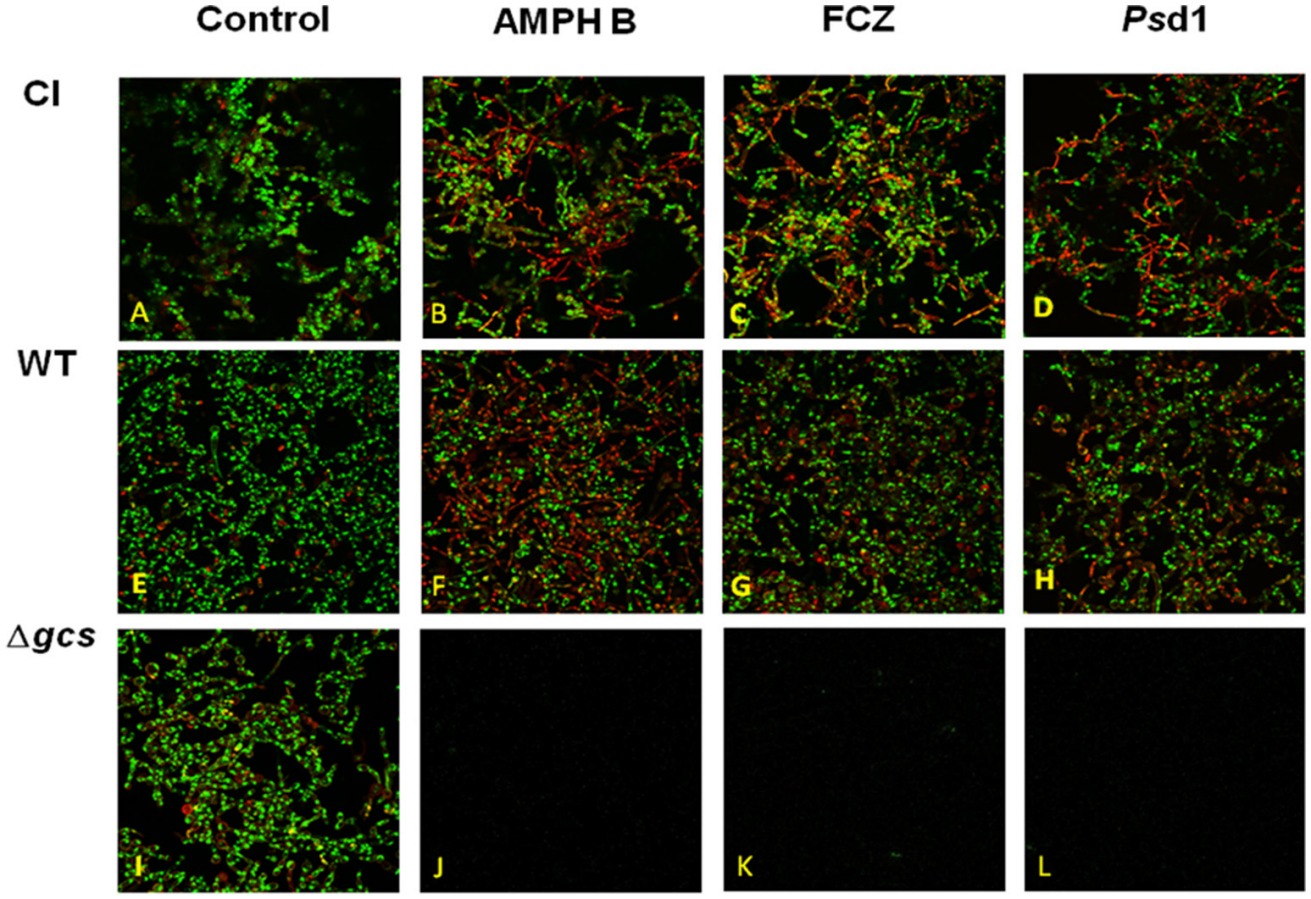
Eradication assays. Confocal microscopy images of the *C*. *albicans* strains clinical isolate **(A–D)**, wild type **(E–H)** and Δ*gcs* **(I–L)**, in the absence **(A,E,I)** and presence of AMPH B **(B,F,J)**, FCZ **(C,G,K)** or *Ps*d1 **(D,H,L)**, at a concentration 10-fold higher than the MIC.

To evaluate the viability of yeast cells before and after antifungal treatments, confocal laser scanning microscopy images were acquire in three biofilm “phases”: formation, inhibition and eradication of the formed biofilm. As shown in Figure 6, the biofilm formed for all strains presented a 3D architecture, consisting of a network of hyphae and budding yeast cells connected at several points. Upon quantification, it was shown that the density of dead cells once the biofilm was formed was low (approximately 10%) for all strains (Figures 6A,E,I). For the inhibition assays, no cells growth was observed after antifungal treatment. Our data indicate that AMPH B, FCZ, and *Ps*d1 inhibited biofilm formation by reducing the rate of its development.

For eradication assays, once the biofilm was developed for each strain the antifungal was added at concentration of 10 × MIC, and incubated for 24h. The results obtained show that the biofilm formed by Δ*gcs* strain was completed eradicated (Figure 6), contrary to CI (Figures 6B–D) and WT (Figures 6F–H), where the architecture of the biofilm suffered perceptible/noticeable changes and the amount of dead cells was increased, especially for CI treated with AMPH B (Figure 6B) and FCZ (Figure 6C), and for WT treated with AMPH B (Figure 6F).

## Discussion

Some *Candida spp*. strains are becoming resistant to the most common antifungal medications. In this sense, many efforts have been made in order to create antimicrobial agents that act along the immune system to eradicate the infection *in vivo*. *Ps*d1 is an AMP with antifungal effects against *C*. *albicans*. In order to understand the mode of action of this peptide, three *C. albicans* strains were studied, one of them, with a mutation in the GlcCer synthase gene, as well as its wild type counterpart. In summary, *Ps*d1 caused important morphological changes, namely at the cell surface, and cell death. Adherence assays of *C. albicans* mutant to abiotic surface enhanced the importance of GlcCer in *Ps*d1 antifungal activity through the study of the strain deficient in GlcCer (Lobo et al., 2007). In fact, some of the observations reported in this work are compatible with these evidences that *Ps*d1 effects rely on the presence of this lipid in the membrane of *C. albicans* (Tyagi and Malik, 2010a; de Medeiros et al., 2014; Rollin-Pinheiro et al., 2016).

AMPs have been tested for their ability to affect physical properties of cells, such as morphology, size, height, roughness, and stiffness (Canetta et al., 2006; Tyagi and Malik, 2010a,b; Kim et al., 2011; Eaton et al., 2012; Alsteens et al., 2013a,b; El-Kirat-Chatel et al., 2013; Formosa et al., 2013). Importantly, *C. albicans* cell wall composition can also be changed upon antifungal treatment, inclusively at the level of the expression of adhesion proteins, therefore also affecting cell-cell interactions (El-Kirat-Chatel et al., 2013; Formosa et al., 2013). Two non-peptidic conventional antifungal agents (AMPH B and FCZ) and a natural AMP (*Ps*d1) cause morphological alterations in *C*. *albicans* (Alviano et al., 1999). Ergosterol is an essential component of the fungal cell membrane. Its direct binding (AMPH B) (Gray et al., 2012) or the inhibition of its synthesis (FCZ) (Chang et al., 2008, 2014) results in increased cellular permeability, causing leakage of cellular contents. Changes observed for *C. albicans* strains occurred at low antifungal concentration and in a short incubation time: a decreased in cell volume, the appearance of blebs or a peeling effect at the cell surface, the increase of its roughness (Tyagi and Malik, 2010a,b) and a lower ability of cells to adhere to each other (Alsteens et al., 2013a,b). These were common features displayed by the action of AMPH B, FCZ, and *Ps*d1. As observed for *Ps*d1 (Figure 4), a peeling-like surface pattern seemed to be an effect frequently induced on *C. albicans*. The results presented here are in agreement to the reported effects induced in *C. albicans* cells by AMPH B, flucytosine (Kim et al., 2011) and caspofungin (El-Kirat-Chatel et al., 2013; Formosa et al., 2013; Hasim et al., 2016).

Among the most commonly accepted models that describes the mode of action of AMPs (Silva et al., 2014), the carpet model is the one that may better explain the effects that causes a larger decrease in membrane homogeneity and, due to a detergent-like micellization, a loss in membrane resistance (Chang et al., 2008). A weakened membrane could suffer disruption, leading to the leakage of cellular contents, explaining the volume loss observed on some cells (Figure 4). By observing the error signal images, it was also possible to notice that *Ps*d1-treated cells did not aggregate like the control cells, or even like AMPH B or FCZ-treated cells. This may evidence that *Ps*d1 has some effect on cell-cell adhesion. This outcome may be explained by the destabilization introduced in the cell wall by *Ps*d1 (El-Kirat-Chatel et al., 2013; Hasim et al., 2016) and by a possible detergent-like action at the membrane level (Da Silva and Machado, 2012).

*C*. *albicans* planktonic cells presented a significant increase in surface roughness after treatment with AMPH B, FCZ or *Ps*d1. Upon surface analysis of AFM height images (Tyagi and Malik, 2010a,b; Domingues et al., 2013; Franquelim et al., 2013), it was possible to quantify the increase in membrane roughness that had been noticed on the error signal images. The fact that Δ*gcs* cells were less affected by these substances may be due to the fact that they are already rougher (Figure 2G) than CI and WT strains, even before treatment with AMPH B, FCZ or *Ps*d1.

The morphological changes due to the action of external agents may also be associated with alterations on cell rigidity (El-Kirat-Chatel et al., 2013; Hasim et al., 2016). AFM-based force spectroscopy measurements allowed the quantification of cell elasticity through Young's modulus calculation (Domingues et al., 2013; El-Kirat-Chatel et al., 2013; Franquelim et al., 2013). *C. albicans* loses stiffness after treatment with AMPH B, FCZ or *Ps*d1. Δ*gcs* cells presented an 18% reduction of the average cell stiffness, comparing to its WT counterpart (Figure 3). This may be due to the lack of GlcCer (Thevissen et al., 2004, 2012; Nimrichter and Rodrigues, 2011). Ceramides are known to increase membranes rigidity, stability and structural organization of biological membranes (Sullan et al., 2009). When compared to AMPH B or FCZ effects in cell surface stiffness reduction, *Ps*d1 had a stronger effect, both on CI and on WT cells, with decreases of 67% and 57% relative to the control sample, respectively (Figure 3). The fact that *Ps*d1 causes a substantially lower decrease on the stiffness of Δ*gcs* cells surface (34%) can be related with the strong evidence that this defensin has glucosylceramide as a molecular target in *C. albicans* cell membrane, as previously suggested (de Medeiros et al., 2010, 2014; Gonçalves et al., 2012; Rollin-Pinheiro et al., 2016). The AMPH B-driven strong reduction of Δ*gcs* cells' stiffness (60%, Figure 3) may be due to a synergistic effect between the pores formed by this antifungal drug at cell surface and the lack of GlcCer in the cell membrane. As the binding of AMPH B to ergosterol is irreversible (Filippin et al., 2008), together with GlcCer absence in *C. albicans* cells, there is no way for the cell to repair the damage caused by antifungal action in the cell surface, becoming unstable and with a lower resistance (Leipelt et al., 2001).

Looking at AFM phase contrast images of *C. albicans* after treatment with *Ps*d1 (Figure S7), it was possible to observe softer domains on its surface, which coincide with the localization of blebs seen in error signal images. These softer areas are probably an effect due to the accumulation of the peptide on the cell surface, eventually acting in a detergent-like manner, leading to a disorganization and micellization of the lipids and, consequently, to unstable cell membrane and wall.

The ability of an antimicrobial peptide to induce cell death is associated with the mechanism by which it acts against its target(s), often at the level of the cell wall and/or membrane. The importance of the type of membrane that the peptide finds is significant; thus, differences between strains would be expected. For CI cells, none of the molecules tested here (AMPH B, FCZ, and *Ps*d1) caused a significant loss of viability (Figures 4B,C and Figure S3), which could only be explained by the mode of action of the peptide in these cells. Even in the membrane, patient cells could have some small biochemical changes in the GlcCer molecules or on the orientation in the membrane, affecting the mechanism of action of the peptide (Rollin-Pinheiro et al., 2016). Although, it was clear from other results that the peptide acts in these type of cells, reducing the roughness and stiffness of the cells (Figures 2, 3, respectively), it is not mandatory to this AMP to induce death or to permeabilize the cell membrane, explaining why there is no positive results for dead cells.

*Ps*d1 had a stronger effect on WT *C. albicans* than the conventional antifungal molecules (AMPH B and FCZ), independently of the concentration used. Aditionally, it was notable that the percentage of dead cells was higher for WT than Δ*gcs* cells (Figures 4D,F,H,I) (de Medeiros et al., 2014). The lack of GlcCer in the membrane of Δ*gcs* cells could explain previous observations regarding this lipid, as it plays an essential role in fungal virulence (Thevissen et al., 2003, 2012; Barreto-Bergter et al., 2004). Although, it is not clear which mechanism predominates for each strain, the reported explanations of the mode of action of individual defensins include binding to the cell wall, induction of signaling cascades and interaction with intracellular targets, leading to apoptosis, membrane permeabilization and receptor-mediated internalization (Thevissen et al., 2004; de Medeiros et al., 2010; Van Der Weerden et al., 2013). Considering that *P*sd1 interacts with *N. crassa* cell cycle protein cyclin F and halts the cell cycle (Lobo et al., 2007), recently, the same has been reported for *C. albicans*, entering in the cells and interacting with an intracellular target, leading to cell death (de Medeiros et al., 2014).

We believe that *Ps*d1 first acts at the fungal wall level, disaggregating the polysaccharide matrix and disturbing the wall, composed by mannoproteins, β-glucans, and chitin. This affects the integrity of the cell wall by increasing cell roughness and decreasing its rigidity. When the peptide reaches the cell membrane, it interacts with glucosylceramides in the *C*. *albicans* membrane and induces an intracellular effect. Later on, the intracellular accumulation of *Ps*d1 interferes with the cell cycle control protein cyclin F, as previously described for *C. albicans* (de Medeiros et al., 2014), in a similar way to what was found for *N. crassa* (Lobo et al., 2007), which leads to apoptosis of the fungal pathogen.

The ability of planktonic cells to adhere to an abiotic surface and to other cells is an important virulence factor, and it is especially important for biofilm formation. By hindering this ability, *Ps*d1 testing against *C. albicans* biofilms becomes highly relevant. Biofilms exhibit increased drug resistance compared to planktonic cells (Figures 1, 5, respectively). Changes in the mode of action of some antifungic molecules vary from planktonic to sessile cell states (biofilm). This is true for AMPH B, FCZ, and *Ps*d1 on *C*. *albicans* biofilms (Figures 5, 6). The complex structure of the biofilm, surrounded by substances rich in exopolymers, with high components of carbohydrates, proteins, hexosamines, phosphorus and uric acid, may restrict the penetration of external agents, decreasing their effects (Figures 5, 6) and increasing biofilm life-time.

This work elucidates some aspects of the *Pisum sativum* defensin 1 antifungal activity that had not been previously investigated. By hindering cells ability to adhere, *Ps*d1 may also contribute to preventing an infection to proliferate, by reducing the adherence of *C. albicans* cells to the infected tissue. It is also possible to consider that *Ps*d1 may interfere with biofilm formation, which depends highly on cell-substrate and cell-cell adherence (Alsteens et al., 2013a,b), as well as on quorum sensing (Kruppa, 2009). Altogether, the ability of morphological forms (fungal pleomorphism) of *C. albicans* was interfered by *Ps*d1 (Figures 1, 5).

The molecular design and synthesis of new molecules inspired on AMPs structure and sequence seem to be a promising approach to open a new and extensive field of applications (Oren et al., 1997; Wu et al., 2003; McPhee et al., 2005), ranging from antimicrobial therapy, to their possible use as vaccine adjuvants. Therefore, a better understanding of function and mechanism of action of host defense peptides is a great promise in anti-infective and immunomodulatory therapeutics. Our previous work showed that *Ps*d1 interacts with membranes which composition mimicking fungal membranes (Gonçalves et al., 2012). Strong interactions with ergosterol and GlcCer-containing membranes were reported, while no interaction with cholesterol-containing membranes justifies a reduced toxicity to mammalian cells (with cholesterol-rich membranes). Here, we show that *Ps*d1 has a strong and pleiotropic antifungal activity on *C*. *albicans*, and the importance of GlcCer as a key component of fungal membranes was underlined.

## Author contributions

Conceived and designed the experiments: SG, Ld, EK, and NS. Performed the experiments: SG, PS, MF, and Ld. Analyzed the data: SG, PS, MF, and Ld. Wrote the paper: SG, PS, MF, Ld, EK, and NS.

### Conflict of interest statement

The authors declare that the research was conducted in the absence of any commercial or financial relationships that could be construed as a potential conflict of interest.
