# Supplementary material for: Psd1 Effects on Candida albicans Planktonic Cells and Biofilms
Source: Front Cell Infect Microbiol. 2017 Jun 9;7:249. doi: 10.3389/fcimb.2017.00249 (PMC5465278; doi:10.3389/fcimb.2017.00249)
Supplement: Supplementary file 1 [file Presentation1.pdf]

Supplementary Material

*Ps*d1 effects on *Candida albicans* planktonic cells and biofilms

Sónia Gonçalves[a,*], Patrícia M. Silva[a], Mário R. Felício[a], Luciano Neves de Medeiros[b], Eleonora Kurtenbach[b], Nuno C. Santos[a,*]

[a] Instituto de Medicina Molecular, Faculdade de Medicina, Universidade de Lisboa, Lisbon, Portugal

[b] Instituto de Biofísica Carlos Chagas Filho, Universidade Federal do Rio de Janeiro, Rio de Janeiro, Brazil

* Corresponding authors:

*Sónia Gonçalves, Instituto de Medicina Molecular, Faculdade de Medicina, Universidade de Lisboa, Av. Prof. Egas Moniz, 1649-028 Lisbon, Portugal. Tel.: +351 217999476; fax: +351 217999477; e-mail: sabreu@fm.ul.pt .

*Nuno C. Santos, Instituto de Medicina Molecular, Faculdade de Medicina, Universidade de Lisboa, Av. Prof. Egas Moniz, 1649-028 Lisbon, Portugal. Tel.: +351 217999480; fax: +351 217999477; e-mail: nsantos@fm.ul.pt.

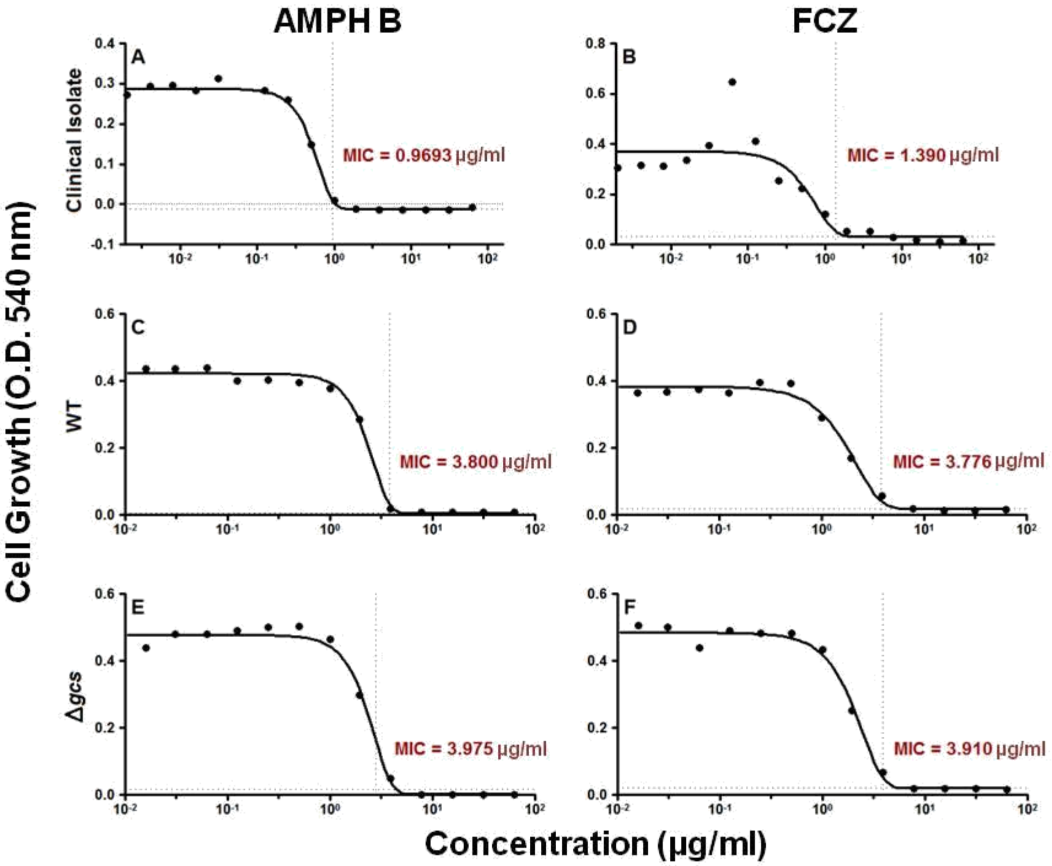

Figure S1. Susceptibility curves of *C. albicans* clinical isolate, wild type and Δ*gcs* planktonic cells to AMPH B and FCZ. Curves were fitted and the MIC values obtained using the Gompertz equation.

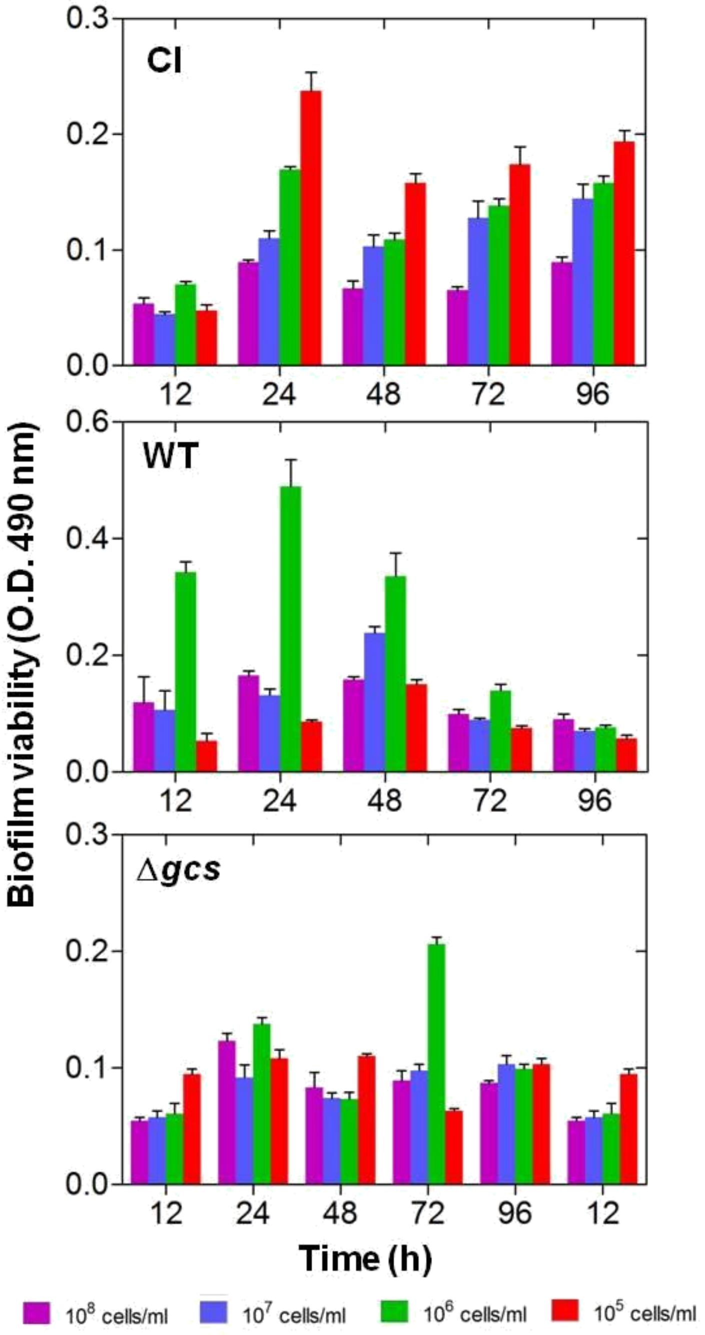

Figure S2. Colorimetric readings from XTT/menadione assays of biofilms formed by the three *C. albicans* strains. Each column represents the mean ± SD from eight independent biofilm readings.

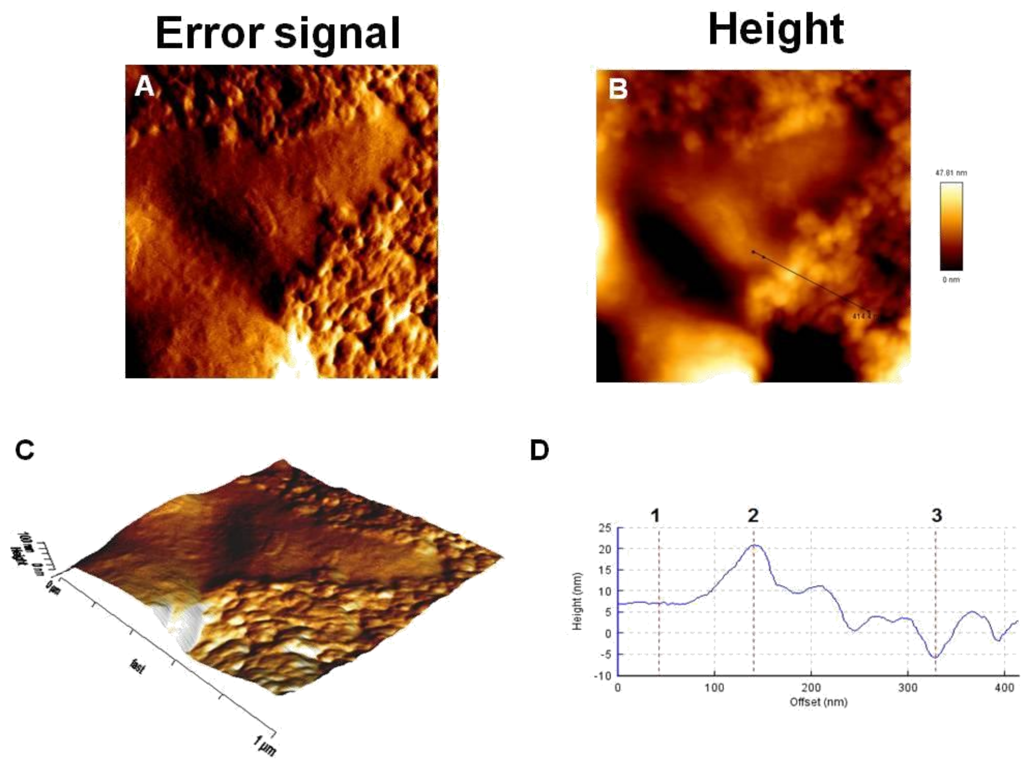

Figure S3. Detail of the blebs on a *C. albicans* wild type cells, after 24 h incubation with *Ps*d1 at 10 × MIC. Error signal (A) and height images (B) are 1 × 1 μm^2. Membrane blebs are easier to depict on a pseudo-3D representation of the cell surface (C). A cross-section of image B (black line) is presented as height profile (D). It enables the determination of a bleb height: the difference in height between 1 (basal cell surface level) and 2 (top of the first bleb in the cross section) is 13.8 nm.

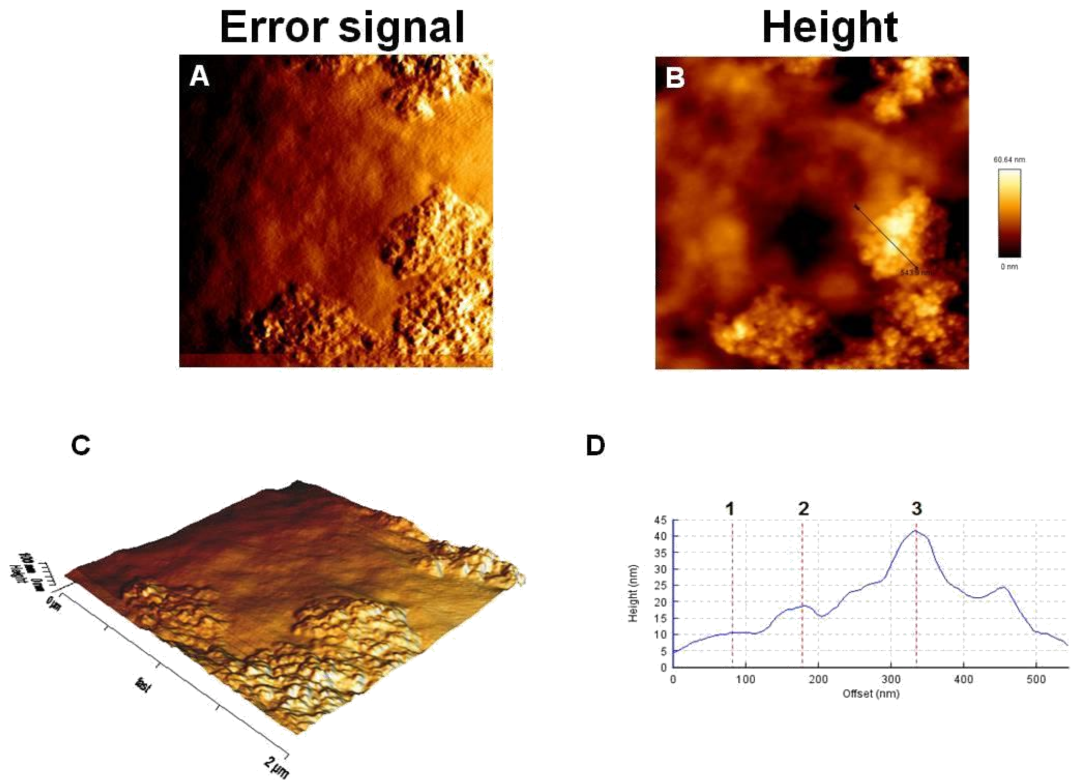

Figure S4. Detail of the blebs on a *C. albicans* Δ*gcs* cells, after 24 h incubation with *Ps*d1 at 10 × MIC. Error signal (A) and height images (B) are 2 × 2 μm^2. Membrane blebs are easier to depict on a pseudo-3D representation of the cell surface (C). A cross-section of image B (black line) is presented as height profile (D). It enables the determination of a bleb height: the difference in height between 1 (basal cell surface level) and 2 (first bleb in the cross section) is 7.8 nm.

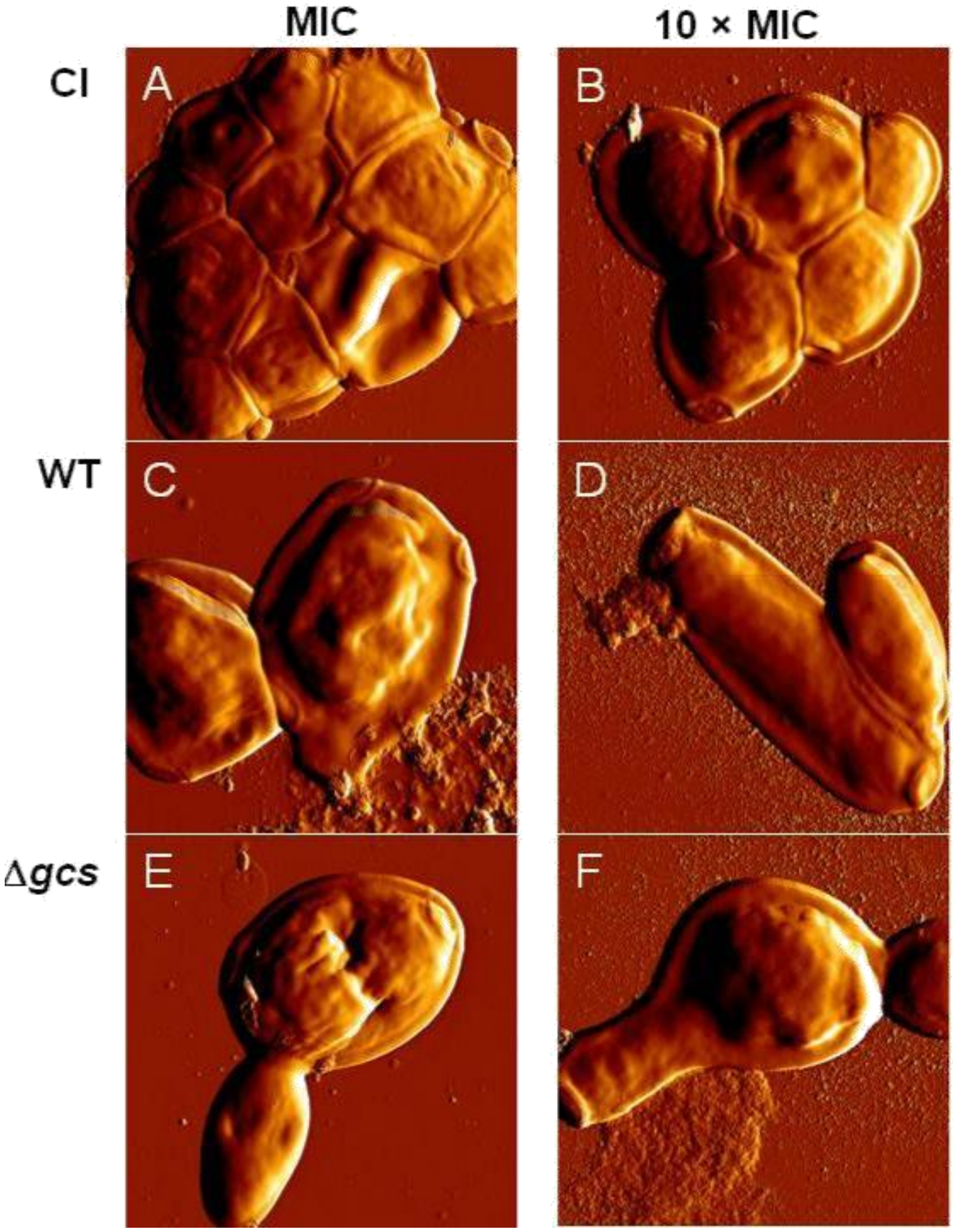

Figure S5. Effect of AMPH B concentration on *C. albicans* planktonic cells. AFM error signal images of clinical isolate (A, B,), Wild type (C, D) and Δ*gcs* (E, G) cells after 24 h incubation with AMPH B. All images are 10 × 10 μm^2.

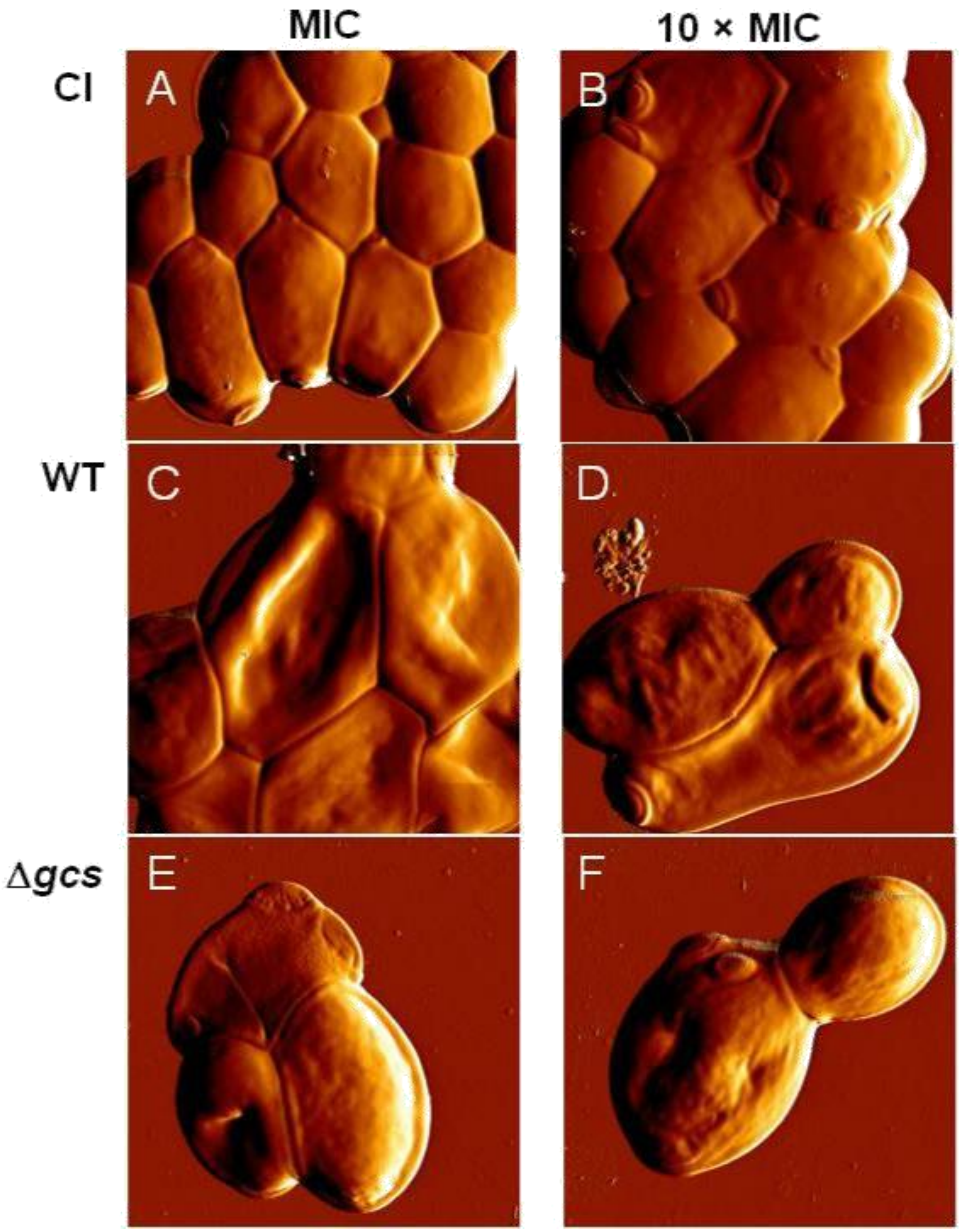

Figure S6. Effect of FCZ concentration on *C. albicans* planktonic cells. AFM error signal images of clinical isolate (A, B,), wild type (C, D) and Δ*gcs* (E, G) cells after 24 h incubation with FCZ. All images are 10 × 10 μm^2.

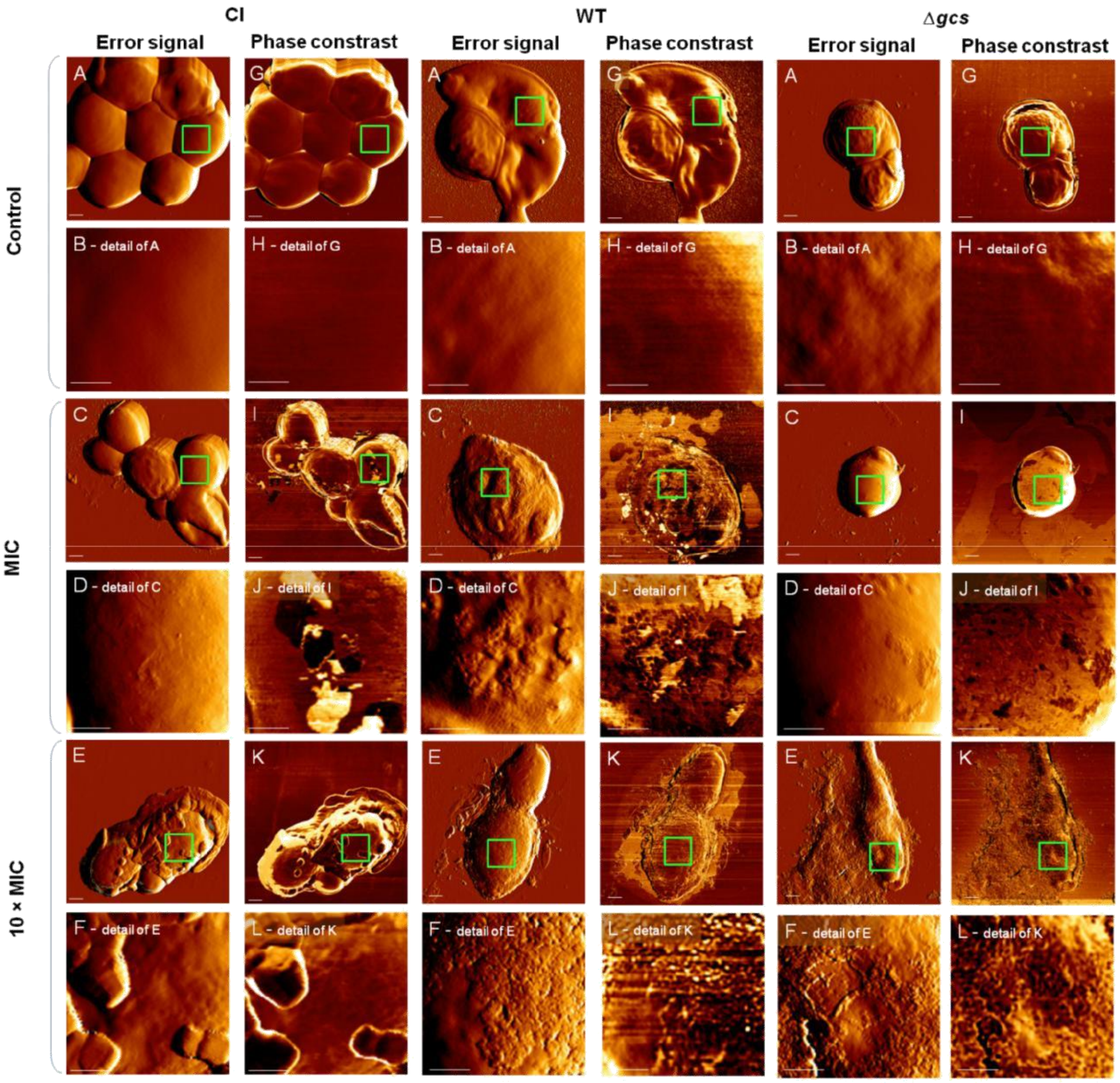

Figure S7. Error signal and phase contrast images of clinical isolate, wild type and Δ*gcs* planktonic cells after 24 h of incubation with *Ps*d1, at MIC and at a 10-fold higher concentration. Details of blebs, peeling and membrane disruption are indicated in C, H, E and J (1 × 1 μm^2). Scale bars correspond to 500 nm.

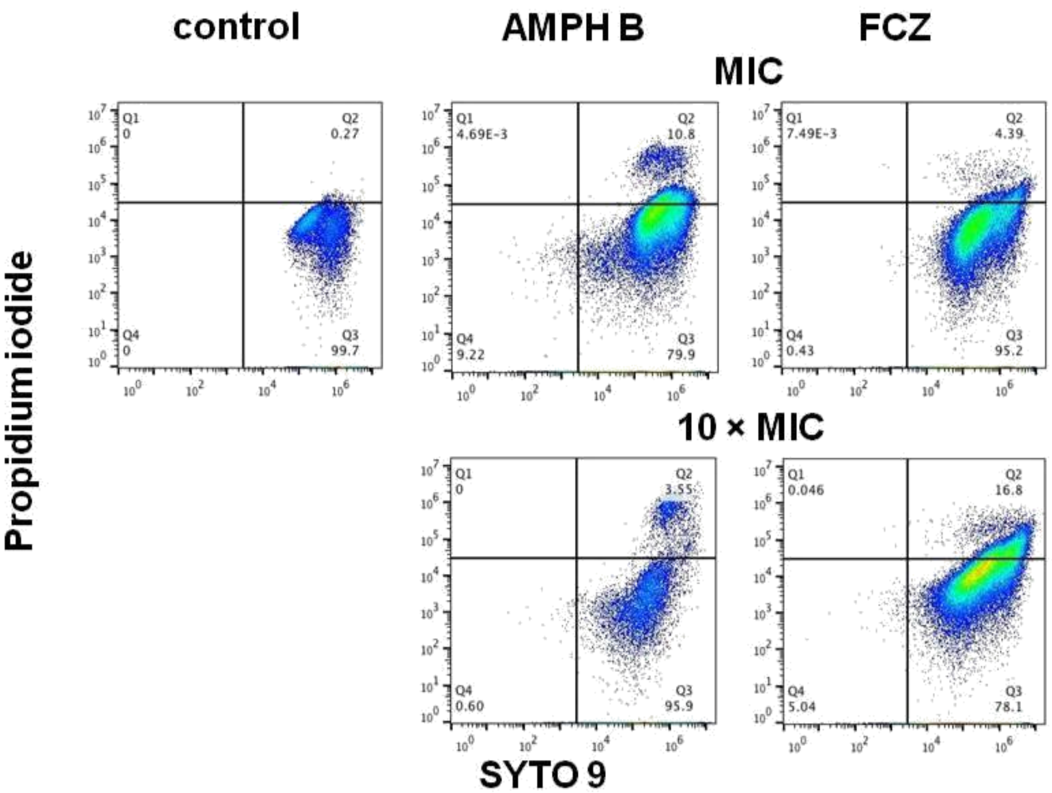

Figure S8. Flow cytometry dot plots of clinical isolate planktonic cells after treatment with AMPH B and FCZ. Cells stained with both dyes after 24 h incubation with antifungals at MIC and 10-folder higher concentration.

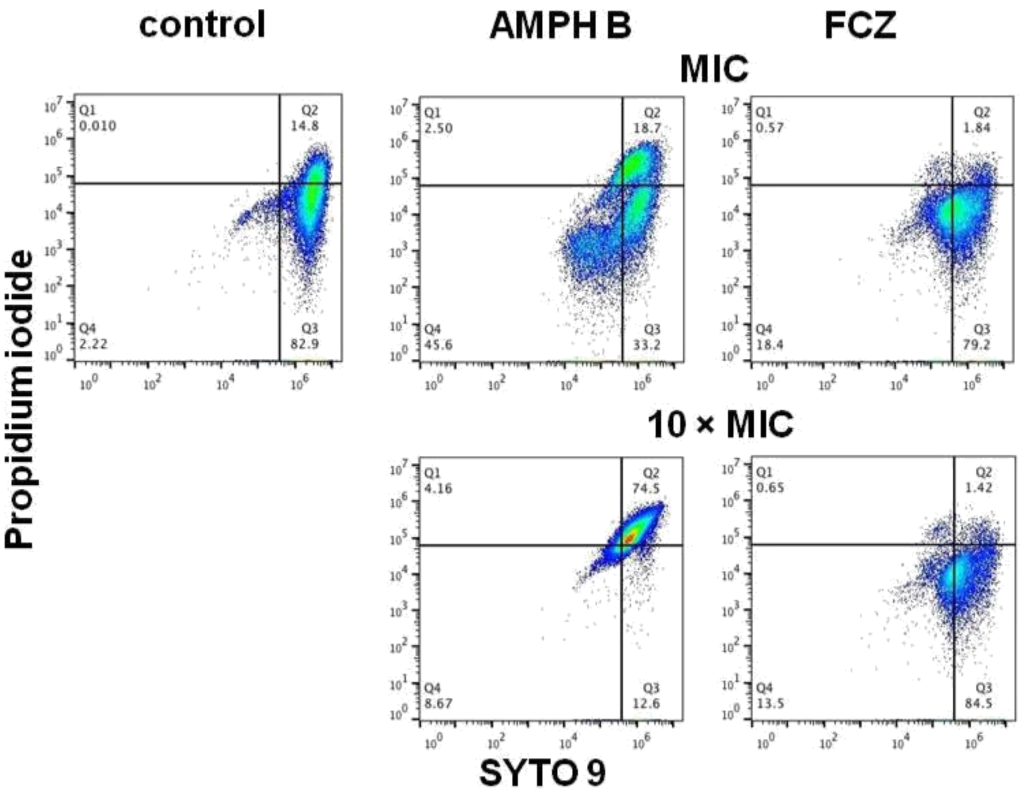

Figure S9. Flow cytometry dot plots of wild type planktonic cells after treatment with AMPH B and FCZ. Cells stained with both dyes after 24 h incubation with antifungals at MIC and 10-folder higher concentration.

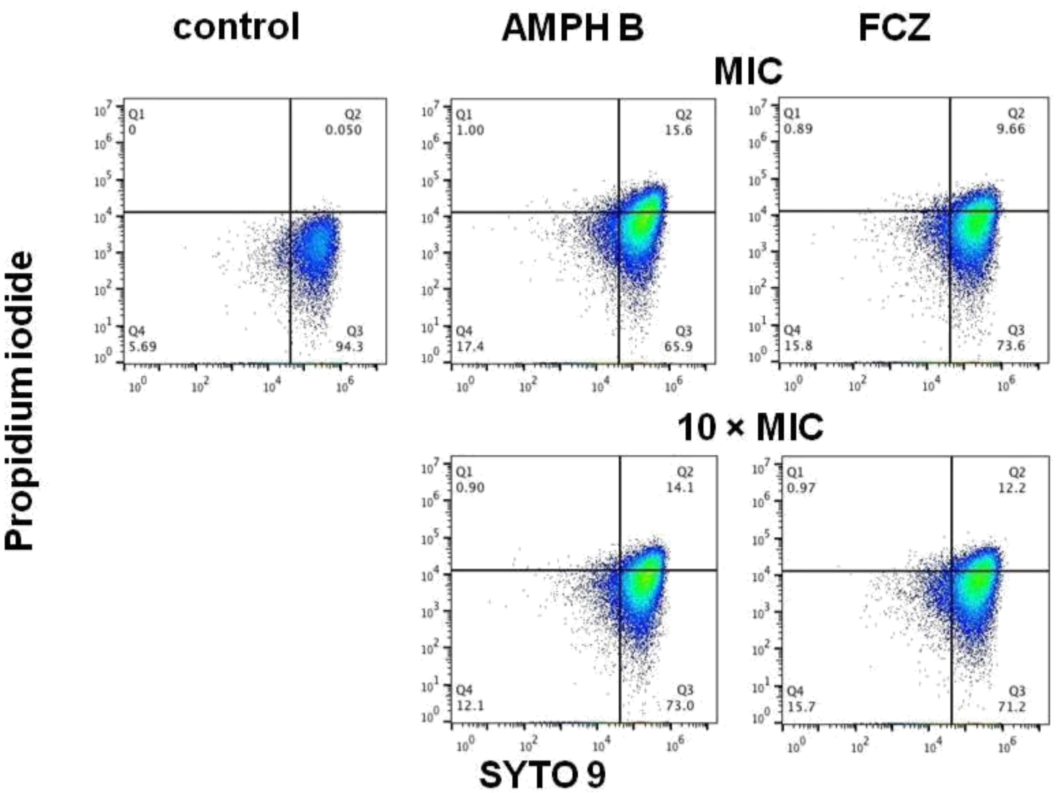

Figure S10 Flow cytometry dot plots of Δ*gcs* planktonic cells after treatment with AMPH B and FCZ. Cells stained with both dyes after 24 h incubation with both antifungals at MIC and 10-folder higher concentration.

Table S1 Biofilm eradication assays. Percentage of live cells after antifungal action.

	**Control**	**AMPH B**	**FCZ**	***Ps*d1**
Clinical isolate	86.5	39.0	35.0	53.4
Wild type	87.8	42.9	84.6	65.7
Δ*gcs*	95.2	-	-	-
